# Systemic thymulin overexpression mitigates cognitive and molecular impairments in a rat model of sporadic Alzheimer’s Disease

**DOI:** 10.1002/alz.089851

**Published:** 2025-01-03

**Authors:** Facundo Peralta, Ana Abril Vidal Escobedo, Juliette López Hanotte, Joaquín Pardo, Paula Cecilia Reggiani

**Affiliations:** ^1^ National Council of Scientific and Technical Research (CONICET/UNLP), La Plata Argentina

## Abstract

**Background:**

Sporadic Alzheimer’s Disease (sAD) is the most prevalent progressive neurodegenerative disease worldwide, without a cure. We propose to investigate therapies that contribute to the current state of this problem using a model of sAD in rats based on a single intracerebroventricular (icv) injection of streptozotocin (STZ). In this sense, thymulin (originally known as serum thymic factor, FTS), a thymic peptide, emerges as a potential therapeutic agent due to its proven anti‐inflammatory effects. Since the cerebral hippocampus (hc) is one of the key vulnerable areas in sAD, our study focuses on the analysis of this region.

**Method:**

We used an adenoviral vector (RAd‐FTS) for systemic thymulin overexpression via intramuscular (IM) injections. Animals were divided into 3 groups (n = 8): SHAM, STZ and STZ+FTS. Rats received bilateral icv injections of artificial cerebrospinal fluid (SHAM) or STZ (STZ and STZ+FTS groups) (3 mg/kg). At weeks 1 and 6 post‐STZ, STZ+FTS animals received IM injections of RAd‐FTS. Prior to sacrifices (week 13), behavioural tests were performed to evaluate species‐typical, exploratory, anxious and depressive behaviours, and recognition memory. At the tissue level, immunohistochemical staining for immature neurons, microglia and astrocytes were performed.

**Result:**

A significant deterioration in all assessed behaviours was observed in the STZ group. However, the STZ+FTS group did not show significant differences compared to the SHAM group in species‐typical, exploratory, anxious, and depressive behaviours. Moreover, it restored the recognition memory that was impaired in STZ animals. At the tissue level, systemic expression of FTS did not ameliorate the impact of STZ on immature neurons but showed an effect on microglia and astrocytes in the hc.

**Conclusion:**

We explored a minimally invasive therapeutic strategy that allowed us to fully or partially reverse behavioural and molecular changes in our animals with sAD. Therefore, our results are encouraging and suggest that thymulin administration may be a promising therapeutic approach, warranting consideration in sAD studies.

